# Research on the matching characteristics of the theoretical digging force of a backhoe hydraulic excavator

**DOI:** 10.1038/s41598-022-19976-x

**Published:** 2022-09-28

**Authors:** Zhigui Ren, Jiahao Li, Xiaoping Pang, Jurong Liu, Tianyu Li, Songsong Yu

**Affiliations:** 1 School of Mechanical Engineering, Shaanxi University of Technology, Hanzhong, 723001 China; 2 Shaanxi Key Laboratory of Industrial Automation, Hanzhong, 723001 China; 3 College of Mechanical and Vehicle Engineering, Chongqing University, Chongqing, 400030 China; 4 Guangxi Liugong Machinery Co., LIU Ltd, Liuzhou, 545000 China

**Keywords:** Civil engineering, Mechanical engineering

## Abstract

The theoretical digging force is the maximum digging resistance that an excavator can overcome, which is an important measure of its digging capacity. To study the matching of the digging capacity with the actual demand and the matching of the working device mechanism, a 36.5 t backhoe hydraulic excavator is used as an example to analyse the distributions of the digging resistance under two different normal digging area working conditions and the maximum digging resistance characteristics of the tool. An appropriate digging postures are selected, based on limit digging force and compound digging force models, the theoretical digging forces under the two working conditions are obtained and matched with the measured digging resistance force values and the limiting factors affecting the digging force. The results show that the average percentage of theoretical digging forces greater than the measured digging resistance under both calculation models is 84.06% rather than 100%. The results of different digging methods all indicate that small chamber locking of the boom cylinder is too often the limiting factor for the digging force, resulting in poor matching of the working device. This study provides guidance for the improvement of the theoretical digging force model and the evaluation of the matching characteristics of the working device.

## Introduction

As multifunctional engineering machinery and equipment, hydraulic excavators are widely used in transportation, water conservancy projects, digging and infrastructure construction and other engineering operations. The digging force is an important indicator to measure the digging capacity of a hydraulic excavator and is also the main basis for guiding the optimal design of the working device. The digging force and the digging resistance are a pair of forces and reaction. Therefore, the digging resistance that the excavator overcomes in the actual operation is the digging force that the excavator must exert to complete the digging operation, also known as played by the excavation capacity. The theoretical digging force refers to the maximum digging force that can be exerted by the excavator in a given digging attitude, i.e., the maximum digging resistance that can be overcome. The theoretical digging force is usually calculated to quantify the digging capacity of an excavator, and if the digging resistance required to be overcome by the excavator during operation is greater than the theoretical digging force, this means that the calculated theoretical digging force has lost its role in characterising the digging capacity. Therefore, it is necessary to match the measured digging resistance of the excavator with the theoretically calculated digging force to determine the degree to which the theoretical digging force characterises the excavator's digging capacity and then refine and improve the theoretical digging solution model. In addition, the theoretical digging force is closely related to the design of the layout of the hinge point of the excavator and the matching between the boom, stick and bucket as well as the three sets of hydraulic cylinders. Therefore, this paper focuses on analysing the limitations of the theoretical digging force and proposes a more effective matching solution to improve the digging capacity of an excavator.

Research in the area of digging resistance has focused on early models of bucket–soil interaction based on classical soil mechanics, on Moore–Cullen failure criteria and passive soil pressure theory for retaining walls, and on the development of present-day digging resistance models and simulations. McKyes^1^ gave a complete expression for soil cohesion, adhesion, internal friction, soil–tool interaction friction, the soil accumulation effect, inertial force and other factors related to soil–tool interaction. Park^2^ developed a model reflecting the interaction between the side cutting plate on the bucket and the soil and developed a virtual reality excavator simulator based on this model. Wei^3^ proposed a new bucket–soil interaction model and gave expressions for the tangential and normal forces on the bucket. Wang Tongjian^4^ and Bi Qiushi^5^ proposed a joint simulation using discrete element multibody dynamics (DEM-MBD) to predict the digging resistance based on various operating conditions and simulated and experimented with mechanical digging resistance. Abo-Elnor^6,7^ and other scholars conducted a three-dimensional finite element analysis (DFEA) of the cutting tool–soil interaction process and verified how to correctly set the failure surface in the simulation model when there is a large displacement of the cutting edge and the effects of different geometric models and operating conditions on the cutting forces. However, these studies have adopted certain assumptions about soil conditions and operating patterns, whereas in practice, the digging object is subject to a great deal of uncertainty and complexity and the digging process is dynamic, making the actual digging resistance difficult to predict or measure directly. Therefore, the authors' team has established that the working object is a common tertiary soil mixed with stones, the working condition is a common mounding operation before stopping and trenching in the main digging area, and the digging method is constant free continuous compound digging. A method for calculating the incomplete digging resistance using measured data from the working devices has been proposed; see reference^8^ for details.

Regarding the theoretical digging force, Flores et al.^9^ carried out a workspace analysis of a hydraulic excavator with a positive shovel based on a kinematic converter and solved for the maximum digging force, but their solution did not consider the limitations of machine stability and adhesion, and they calculated only the digging and breaking forces in the characteristic directions. Janosevic^10^ considered the influence on the digging resistance of the active force of the hydraulic cylinder of a hydraulic excavator, the stability of the machine and the possible direction of the digging resistance at the digging point in the working space; calculated the boundary digging forces in different directions in the working space; and connected the ends of the boundary digging force vectors to form a vector polygon and correspondingly presented a modified digging force calculation method. The authors' team^11^ previously established a theoretical digging force calculation method based on the inverse kinematic solution for the digging attitude with the digging point as the object and considering the overall machine stability, ground adhesion and the driving and blocking conditions of each hydraulic cylinder. In addition, based on a study of measured digging resistance characteristics^12^, it was found that the digging resistance was mainly composed of tangential component forces, while the normal component forces and resistive moments also made nonnegligible contributions. The distribution intervals of the ratio of the normal to the tangential component of the digging resistance, called the drag coefficient, and the ratio of the resistance moment to the tangential resistance, called the resistance moment coefficient, were also calculated and found to have a relatively constant distribution interval, called the main value interval, in terms of the directional angle of the digging resistance. Based on a study of the resistance characteristics, the authors developed analytical expressions and solutions for a limit digging force (LDF) model of a bucket and a limit digging force model of a stick, considering the influence of the normal force and the resistance moment on the theoretical digging force^13^. Based on a study of the directional angle of the digging resistance, the authors' team established the main value interval of the directional angle of the compound digging force (CDF) based on the main value interval of the resistance angle and the bucket inverse angle, and they solved for the possible compound digging force in certain steps based on discrete main value intervals, the maximum of which is the compound digging force for a given digging attitude^14^.

Recent research on hydraulic excavators has focused on the study of energy recovery and utilisation in digging operations^15,16^ the study of the energy saving characteristics of the boom and stick of the working device^17,18^ the study of the energy consumption of hybrid excavators^19^, trajectory planning for excavator operations^20,21^, the study of automatic walking based on environmental recognition^22^ and the study of intelligence in autonomous operations^23,24^. Based on the measured digging resistance, this paper analyzes its distribution law and force value characteristics, and selects a suitable digging attitude, based on the bucket and stick limit digging force model and compound digging force model, the theoretical digging force is solved, and the measured digging resistance is matched with the theoretical digging force in size, in order to get the representation degree of the theoretical digging force to the digging capacity. And analyse the limiting factors affecting the application of the digging force and the matching characteristics of the digging work. The matching characteristics of the unit are also analysed.

## Measured digging resistance and selection of digging attitude

### Measured digging resistance

The test subject was a 36.5 t medium-sized backhoe hydraulic excavator excavating tertiary soil mixed with stones at a test site in Huzhou, Zhejiang Province, China. Three NS-RB type angular displacement sensors and six NSF type pressure sensors were used to test the digging attitude and the pressure of rod cavity and rod cavity respectively. Two different conditions of free continuous digging in the normal digging area were selected, where Condition 1 was the operation of normal soil piling on the stopping surface and Condition 2 was the operation of free continuous trenching in the main digging area. Based on the incomplete digging resistance model^8^ established by the team, the angular displacement data required in the tests included the angle of rotation of the boom relative to the body, the angle of rotation of the stick relative to the boom and the angle of rotation of the bucket relative to the stick; the required pressure data included the pressure data of the rod and rodless chambers of the boom hydraulic cylinder, stick hydraulic cylinder and bucket hydraulic cylinder; and the angular displacement and sensor location layout in the tests are detailed in reference^25^.

**Figure 1:**
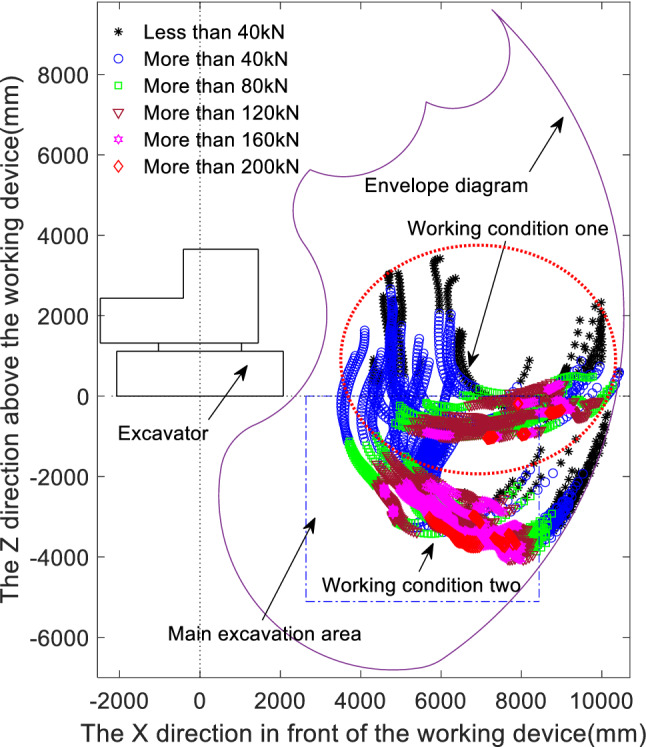
Measured digging resistance distribution diagram.

Figure 1 shows the distributions of the digging points in space for Working Condition 1 and Working Condition 2, where the digging points circled by the dotted circle correspond to Working Condition 1 and the remaining digging points correspond to Working Condition 2, as indicated by the arrows in the figure. The results of dividing the digging resistance at each digging point into intervals of 40 kN are shown in the figure, where the different colours and symbols represent the different digging resistance intervals.

To investigate the distributions of the digging resistance in accordance with the different methods of interaction between the bucket tip and the soil, the digging process can be divided into the stage in which the bucket cuts into the soil to adjust the digging attitude, the stage in which the bucket cuts into the soil for loading, and the stage of rotary lifting of the fully loaded bucket. As shown in Fig. 1, the digging resistances under both conditions are less than 80 kN in the initial bucket cutting stage and the full-load lifting stage, while the digging resistance is greater in the bucket cutting and loading stage and increases with increasing digging depth. Compared with Condition 2, the digging resistance of the soil pile before stopping first gradually increases and reaches a maximum near the maximum digging depth and then gradually decreases to zero at the completion of the digging operation. However, near the maximum digging depth, there are two incomplete operations, as shown in the figure. Near the maximum digging depth, the digging resistance is greater than 200 kN (represented by diamonds); see Fig. 3 later in the manuscript for a locally enlarged view. In the main digging area of the normal digging area, the digging resistance increases instantaneously when the digging depth reaches its maximum at the beginning of the soil cutting phase and when the digging resistance is adjusted near the maximum digging depth. Regardless of the digging conditions, the digging resistance is greatest near the deepest part of the main digging area and when adjusting the digging stance, and depending on the experience of the operator, digging may not be possible during stance adjustment. Therefore, matching the theoretically calculated digging force with the digging resistance in the main digging area and at the deepest point of digging is the focus of this study.

### Selection of digging attitude

**Figure 2:**
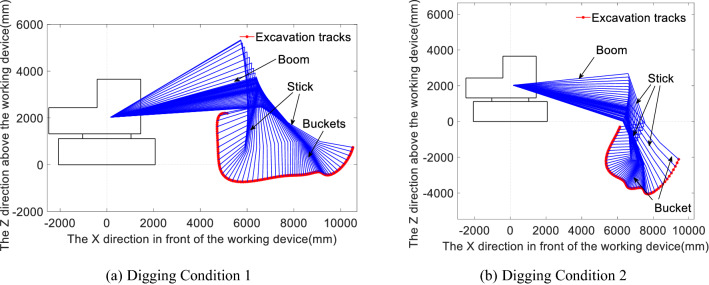
Figures (**a**) and (**b**) show the digging attitudes along the trajectories with the maximum digging resistance under Conditions 1 and 2, respectively.

The digging force and digging resistance form a pair of action and reaction forces. The study of the maximum digging resistance characteristics has important reference significance for perfecting and improving the theoretical digging force model, and the maximum digging resistance is often considered the embodiment of an excavator’s digging capacity. Figure 2a and b show the complete digging operation trajectories and the corresponding operating attitudes at which the maximum digging resistance occurs under the selected working conditions, Conditions 1 and 2, respectively, where the attitudes are obtained at discrete digging points along the digging trajectory separated by a certain step length and equal time intervals. By combining Figs. 1 and 2, i.e., the digging resistance distribution and the digging trajectory attitude reduction diagram it can be found that the denser the spatial locations of the working device are, the greater the digging resistance, the longer the time spent and the slower the operating speed.Therefore, the selection of digging attitude should choose the place with dense digging attitude, where the digging resistance is bigger, and it should be in the main cutting loading stage, which also reflects the digging ability of excavator to overcome the digging resistance.

**Figure 3:**
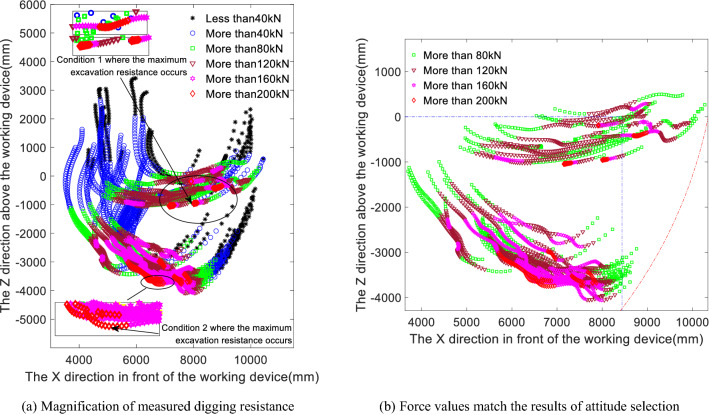
Figures (**a**) and (**b**) show the magnification of measured digging resistance and the force values match the results of attitude selection.

The team modelled the theoretical limit digging force for the bucket-alone digging condition and the stick-alone digging condition, taking into account the effects of normal forces and resisting moments^13^. Referring to the enlarged view of the measured digging resistance shown in Fig. 3a above, the theoretical digging force is calculated as the maximum force that can be exerted by the excavator under a given operating condition with a given digging attitude. Therefore, matching the full digging resistance against the theoretical digging force makes little sense and does not reflect the true characterisation capability of the theoretical digging force. Instead, to ensure comparability and relevance, stages with less resistance should be removed, and only stages with greater digging resistance should be matched against the theoretical digging force while considering the force magnitude and the working device limitations that affect the digging force.

As seen from the previous analysis of the digging resistance distribution, in the initial cutting stage and full-load lifting stage, the digging resistance is small, while in the cutting and loading stage, the digging resistance is larger. Figure 3a shows the distribution of the digging resistances. A digging point with a resistance greater than 80 kN generally corresponds to the cutting and loading stage and thus occurs in a key stage of the digging process, the stage in which the greatest resistance occurs. Therefore, points with digging resistances less than 80 kN are excluded from Fig. 3a, and the remaining digging points form the digging resistance distribution in the cutting and loading stage, as shown in Fig. 3b.

## Theoretical digging force magnitude matching characteristics

### Limit digging force matched against digging resistance

**Figure 4:**
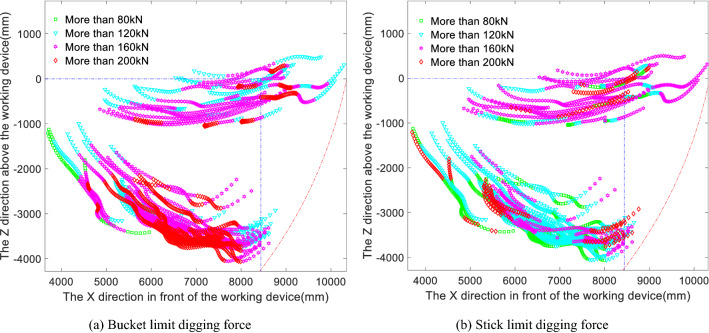
Panels (**a**) and (**b**) show the distributions of the digging points in terms of the bucket limit force and the stick limit force, respectively, during the cutting and loading phase.

The digging points shown in Fig. 3b and the main values of the position, hydraulic cylinder active force, drag coefficient and moment coefficient for each digging point are input into the limit digging force model to obtain the limit digging force distribution of the bucket, as shown in Fig. 4a, and the limit digging force distribution of the stick, as shown in Fig. 4b, along each digging trajectory. The different colours in the diagrams represent the different magnitudes of the theoretical digging forces at the corresponding digging points. By comparing the limit digging force of the bucket with the limit digging force of the stick in Fig. 4a and b, it can be seen that the overall limit digging force of the bucket is greater than that of the stick and that the digging force of the bucket is also significantly greater than that of the stick near the maximum digging depth.

**Figure 5:**
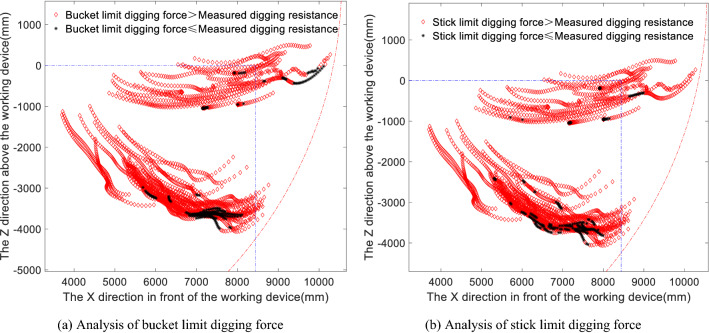
Panels (**a**) and (**b**) show the limit digging forces of the bucket and stick, respectively, compared to the measured digging resistance.

Next, the limit digging forces of the bucket and stick in the cutting and loading phase are matched against the measured digging resistance, the results of which are shown in Fig. 5a for the comparison of the bucket limit digging force and digging resistance and in Fig. 5b for the comparison of the stick limit digging force and measured digging resistance. This analysis shows that the limit digging forces of the bucket and stick both have a measured digging resistance greater than the theoretical digging force and are concentrated at the maximum digging depth and at locations corresponding to the adjustment of the digging posture, which are also the locations where the digging resistance is greater. The digging force of the stick is less than the measured digging resistance, and there are more digging points where the measured digging resistance is greater than the digging force of the stick in Working Condition 2 than in Working Condition 1; thus, it can be seen that if the stick digs alone, no digging may occur at the maximum digging depth. In contrast, the digging points at which the digging force of the bucket is less than the measured digging resistance are more spread out in the cutting and loading phase. The measured resistance exceeds the bucket digging force mostly near the maximum digging depth and, to a lesser extent, in the initial loading phase. To investigate the reasons for this, the proportions of the digging points at which the bucket and stick digging forces do and do not exceed the measured digging resistance are shown in Table 1. Although the actual operation is constant compound digging, the comparison with the theoretical separate digging operations is presented here to reflect that in practice, digging operations may be performed based on a variety of combinations of different components. Accordingly, the specific process of comparing the statistics of the individual operations is demonstrated here in the compound digging force comparison, and the results are given here directly.

**Table 1:** Statistical results for matching the limit digging forces of the bucket and stick against the magnitude of the digging resistance.

Results of matching the limit digging force against the digging resistance	Total percentage share (%)	Factor by which the limit digging force is greater than the measured resistance	Percentage (%)
Bucket limit digging force > measured digging resistance	84.40	More than 1.0–1.1 times	28.23
		More than 1.1–1.3 times	26.70
		More than 1.3–1.5 times	16.34
		More than 1.5–1.7 times	10.33
		More than 1.7 times greater	18.40
Bucket digging force ≤ measured digging resistance	15.60	Factor by which the measured resistance is greater than the limit digging force	Percentage
		More than 1.0–1.1 times	94.58
		More than 1.1 times greater	5.42
Stick digging force > measured digging resistance	81.58	Factor by which the limit digging force is greater than the measured resistance	Percentage
		More than 1.0–1.1 times	32.50
		More than 1.1–1.3 times	21.40
		More than 1.3–1.5 times	18.56
		More than 1.5–1.7 times	11.30
		More than 1.7 times greater	16.24
Stick digging force ≤ measured digging resistance	18.42	Factor by which the measured resistance is greater than the limit digging force	Percentage
		More than 1.0–1.1 times	97.61
		More than 1.1 times greater	2.39

From the analysis of Fig. 5a and b and Table 1, it can be seen that when the bucket digging force is greater than the measured digging resistance or the stick digging force is greater than the measured digging resistance, the digging force is more than 1.1 times the measured digging resistance in a large proportion of instances, indicating that the digging capacity of the excavator is still relatively rich, although this capacity failed to be effectively applied in the actual test. However, for both the bucket digging force and the stick digging force, there are cases in which the measured digging resistance is greater than the theoretical digging force, which is contrary to the definition of the theoretical digging force. The reason may be that in practice, the compound digging force is greater than the sum of the individual bucket digging force and stick digging force. For the 15.6 and 18.42% of cases in which the measured digging resistance is greater than the bucket digging force and the stick digging force, respectively, the factor by which the two force values differ is in the range of 1–1.1 94.5 and 97.61% of the time, respectively, while this factor is greater than 1.1 in only 5.42 and 2.39% of instances, respectively. These findings show that although the measured digging resistance is greater than the theoretical digging force, the difference tends to be quite small, and the calculation result is acceptable. The reason for this may be that the values of the drag and moment coefficients in the limit digging force model tend to remain within a limited range of values rather than spanning the entire possible range.

### Compound digging force matched against digging resistance

**Figure 6:**
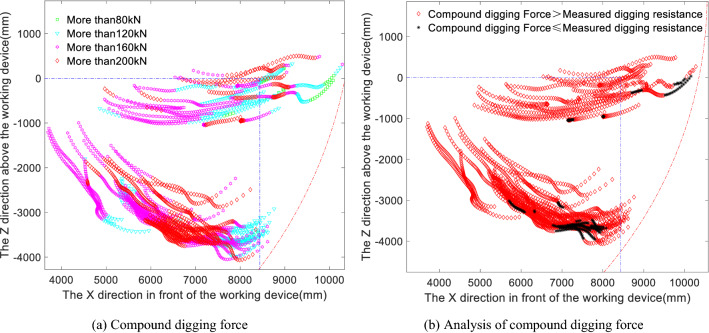
(**a**) Distribution of digging points in terms of the compound digging forces during the cutting and loading phase. (**b**) Compound excavation force compared to measured excavation resistance during the cutting and loading phase.

the stick hydraulic cylinder in conjunction with each other. The same digging resistance trajectory points and their test data from the cutting and loading phase shown in Fig. 3b were then input into the compound digging force calculation model to obtain the compound digging force distribution diagrams shown in Fig. 6a.

Comparing the compound digging force in Fig. 6a with the bucket and stick limit digging forces in Fig. 4a and b reveals that the distribution of the compound digging force is similar to that of the bucket digging force, in that the trajectory of the digging points with a digging resistance greater than 160 kN is similar to that of the digging force when the digging depth is adjusted and the digging attitude is adjusted. Regarding the distribution of points with digging resistances greater than 200 kN, the compound digging forces at the corresponding digging points are higher than the bucket digging forces, which shows that the digging performance of the compound digging force is better than that of the bucket digging alone and the stick digging alone. When the compound digging force is compared with the bucket digging force, the overall magnitude of the compound digging force distribution is greater than that of the bucket digging force distribution. Now, the compound digging force distribution is matched against the measured digging resistance, i.e., the compound digging force in Fig. 6a is compared with the measured digging resistance distribution in Fig. 3b, and the matching results are shown in Fig. 6b.

**Figure 7:**
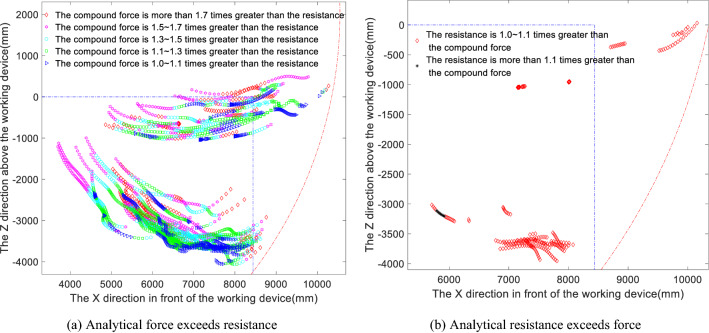
(**a**) Analysis of the proportion of instances in which the compound digging force is greater than the measured digging resistance. (**b**) Analysis of the proportion of instances in which the measured digging force is greater than the compound digging force.

An analysis of Fig. 6b shows that for the compound digging force, a situation also appears in which the measured digging resistance is greater than the compound digging force. By combining Figs. 5a, b and 6b, i.e., comparing the limit digging force, the compound digging force and the measured digging resistance, it can be seen that the points at which the measured digging resistance is greater than the compound digging force and the limit digging force of the bucket in the cutting and loading stage are concentrated at the beginning of Working Condition 1 and near the maximum digging depth of Working Condition 2. Whereas the measured digging resistance is greater than the bucket digging force mainly in Working Condition 2 in the cutting and loading stage and at the beginning of the full-load lifting stage. Similarly to the limit digging force analysis, a further quantitative analysis of the differences between the two forces is also performed. The proportion of instances in which the compound digging force is greater than the measured digging resistance is shown in Fig. 7a, the proportion of instances in which the measured digging resistance is greater than the compound digging force is shown in Fig. 7b, and the statistical results of these comparisons between the measured digging resistance and the compound digging force are shown in Table 2.

**Table 2:** Statistical results for matching compound digging forces to measured digging resistance magnitudes.

Compound digging force and digging resistance matching results	Total percentage share (%)	Factor by which compound force > measured resistance	Percentage (%)
Compound digging force > measured digging resistance	86.20	More than 1.0–1.1 times	14.00
		More than 1.1–1.3 times	31.40
		More than 1.3–1.5 times	23.60
		More than 1.5–1.7 times	16.30
		More than 1.7 times greater	14.70
Compound digging force ≤ measured digging resistance	13.80	Factor by which measured resistance > compound force	Percentage
		More than 1.0–1.1 times	91.70
		More than 1.1 times greater	8.30

In summary, when comparing Figs. 5a, b and 6b, it can be seen that most of the points at which the measured digging resistance is greater than the theoretical digging force are near the maximum digging depth or when adjusting the digging attitude, i.e., when the digging resistance is the greatest, while a small part are concentrated in the initial bucket cutting stage and at the beginning of the full-load lifting stage. These exceedance regions are similar between the bucket digging condition and the compound digging condition, which also confirms that the bucket digging force and the compound digging force distribution diagrams are similar. When the distributions of the digging points where the measured digging resistance exceeds the limit digging force of the bucket, the limit digging force of the stick and the compound digging force are compared, it can be seen that there are more digging points where the measured digging resistance exceeds the digging force of the stick, indicating that the actual working digging force, i.e., the measured digging resistance, is greater than the theoretically calculated digging force of the stick. This indicates that more often than not, bucket digging and compound digging are used in the vicinity of the measured maximum digging depth. The relatively small distributions of points where the measured digging resistance exceeds the bucket limit digging force or the compound digging force confirm that the bucket digging force and the compound digging force are greater than the stick digging force.

## Theoretical digging force limiting factor matching characteristics

### Characteristics of maximum digging resistance working device

Because the theoretical digging force is calculated when the maximum digging force of the working device constraints. Therefore, in order to analyze the matching characteristics of the working device, we must first analyze the working characteristics of the working device when the maximum digging resistance.Analysis compares the maximum digging resistance trajectory and its force magnitude between Working Condition 1 and Working Condition 2. The average digging resistance and maximum digging resistance under Working Condition 2 are larger than those under Working Condition 1. Notably, a backhoe hydraulic excavator often operates below the stopping surface, and the deeper the depth at which digging is occurring, the more severe the environment. Therefore, the trajectory with the maximum digging resistance under Condition 2 was selected, the data noise at the beginning and end of the operation was removed, and the stable operating interval of the digging trajectory in the test was selected to analyse the real-time variation in the hydraulic pressure of each working device during constant compound digging.

**Figure 8:**
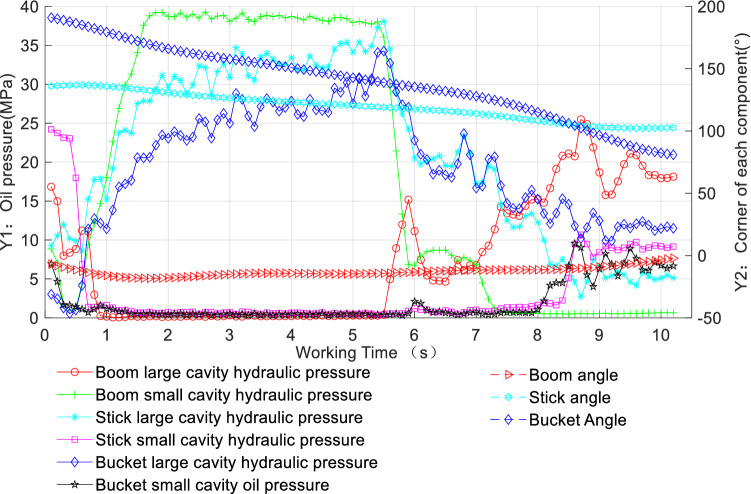
Diagram of the hydraulic cylinder length and the angle of rotation of each component in real time for the trajectory with the maximum digging resistance under Working Condition 2.

Figure 8 shows the real-time oil pressure and the change in the angle of rotation of each component of the working device for the digging trajectory under Working Condition 2 shown in Fig. 2b. By considering Figs. 2b and 8 together, the following analysis can be made. In the initial 0 ~ 2 s, the boom angle gradually decreases to lower the digging position, and the bucket angle begins to decrease to cut into the soil, while the change in the stick angle is not significant. At this time, the oil pressure in the small chamber of the boom increases to the maximum, and the oil pressures in the large chambers of the stick and bucket begin to increase sharply, while the oil pressures in the large chamber of the boom and the small chambers of the stick and bucket remain almost unchanged. From 3 to 6 s, the bucket is mainly loaded by soil cutting, while at approximately 3 and 5.8 s, there are two peaks in the stick and bucket chamber oil pressures, indicating that the moment of the maximum digging force may occur, which is also the moment of the maximum digging resistance, corresponding to the two densest sections of the bucket trajectory in Fig. 2b. After 6 s, the full-load lifting phase is mainly carried out. During this phase, the oil pressure in the small chamber of the boom starts to drop sharply, while the oil pressure in the large chamber of the boom gradually increases and the oil pressures in the large chambers of the stick and bucket gradually decrease in a fluctuating manner, whereas the oil pressures in the small chambers of both the latter components do not change much. After 8.2 s, the stick angle remains almost unchanged, the boom angle continues to increase, and the bucket angle decreases to the minimum to ensure that the soil state is met and the lifting operation is completed. At this time, the oil pressure in the large chamber of the boom reaches the maximum, while the oil pressure in its small chamber is almost 0. The oil pressures in the large and small chambers of the stick and bucket are similar to each other; however, the oil pressure in the large chamber of the bucket is greater than the oil pressure in the small chamber, while the oil pressure in the small chamber of the stick is greater than the oil pressure in the large chamber. Overall, the rate of change of the bucket angle is greater than that of the stick angle, which means that the bucket cylinder plays a greater role than the stick cylinder in compound digging operations.

In summary, in the normal compound digging process, during the cutting and loading stage, the digging resistance is larger; at this time, the oil pressure in the small chamber of the boom is very high, and the small chamber locking capacity requirements of the boom are also high. Because the large chambers of the stick and bucket mainly play the active role of providing hydraulic cylinder thrust, the small chambers of these two components and the large chamber of the boom are almost not involved in the operation. In the initial bucket cutting stage and the full-load lifting stage, the digging resistance is small, and a corresponding investigation of working device matching does not effectively reveal any superiority or inferiority of the excavator design. To meaningfully study the matching characteristics of an excavator’s working device, it is necessary to consider behaviours related to larger digging resistances, i.e., the behaviours of the bucket and stick cylinders in the cutting and loading stage, when the hydraulic pressures in the large chambers are higher. Therefore, the digging attitude shown in Fig. 3b is also used to analyze the matching characteristics of theoretical digging force limiting factors.

### Limit digging force limiting factor matching

**Figure 9:**
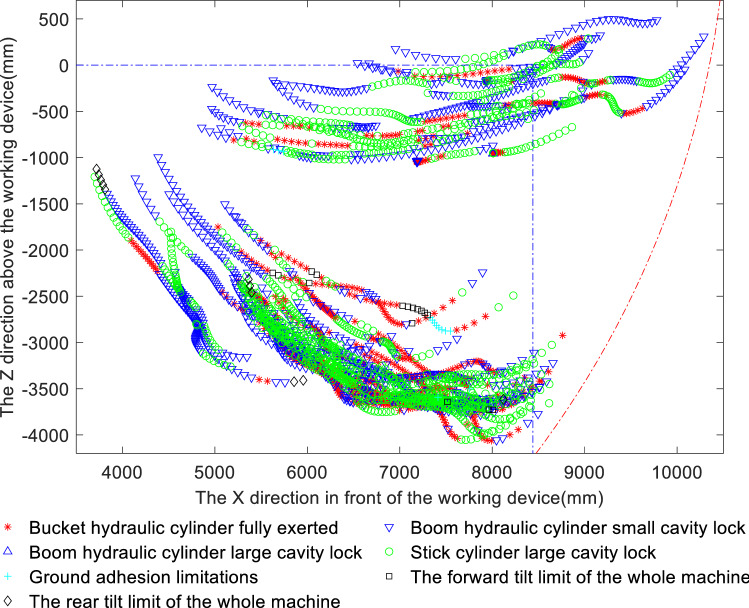
Distribution of limiting factors for the bucket limit digging force.

**Figure 10:**
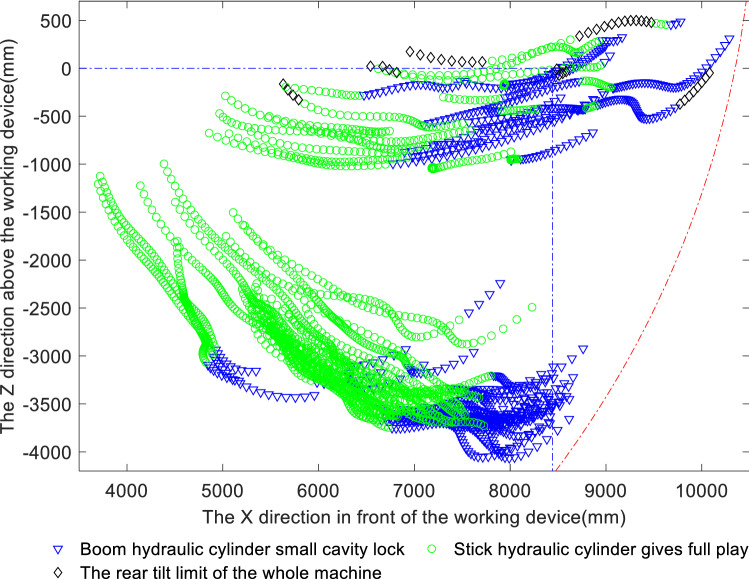
Distribution of limiting factors for the stick limit digging force.

To improve the digging capacity of the excavator and to reduce the impact of the limiting factors affecting the theoretical digging force, the matching characteristics of the various components of the working device are studied in terms of the factors limiting the theoretical digging force in the bucket and stick limit digging force models and the compound digging force model. Based on the above analysis of the maximum digging resistance conditions, it can be concluded that the digging points corresponding to the initial bucket cutting stage and the full-load lifting stage, in which the digging resistance is low, should again be removed from the analysis, while the bucket cutting and loading stage, in which the digging resistance is greater than 80 kN, should be retained. Figures 9 and 10 show the distributions of the limiting factor at each digging point for the bucket limit digging force and the stick limit digging force, respectively, where differently coloured symbols represent different limiting factors. Table 3 provides statistics on the percentages of the digging points limited by each limiting factor.

**Table 3:** Theoretical statistics of the digging force limiting factors.

Limiting factor	Instances in which the bucket limit digging force is limited by the corresponding factor (%)	Instances in which the stick limit digging force is limited by the corresponding factor (%)	Instances in which the compound digging force is limited by the corresponding factor (%)
Bucket hydraulic cylinder in full play	34.16	0	26.20
Small chamber locking of the bucket hydraulic cylinder	–	0	–
Large chamber locking of the bucket hydraulic cylinder	–	0	–
Small chamber locking of the boom hydraulic cylinder	38.12	33.60	34.40
Large chamber locking of the boom hydraulic cylinder	0.34	0	0.15
Stick hydraulic cylinder in full play	0	62.23	37.40
Small chamber locking of the stick hydraulic cylinder	0	–	–
Large chamber locking of the stick hydraulic cylinder	26.22	–	–
Ground adhesion limitation	0.42	0	0.65
Forward tilt limit of the whole machine	0.52	0	0.80
Rearward tilt limit of the whole machine	0.22	4.17	0.40

Analysis of Fig. 9 and Table 3 shows that among the factors limiting the limit digging force of the bucket, the bucket hydraulic cylinder is fully utilised in 34.16% of instances; large chamber locking of the bucket hydraulic cylinder occurs in 26.22% of instances, a relatively high proportion, and small chamber locking of the boom occurs too often, in 38.12% of instances, resulting in the digging force of the bucket hydraulic cylinder not being fully utilised. In comparison, the limitations on the ground adhesion and forward and rearward tilt of the whole machine account for only a small percentage of the impact on digging performance and thus are not significant. Analysis of Fig. 10 and Table 3 shows that although the stick limit digging force is usually well exploited, with the full capacity of the stick hydraulic cylinder being available 62.23% of the time, small chamber locking of the boom limits the digging force in 33.6% of instances, indicating that this phenomenon requires further consideration to improve the digging performance. Figure 10 also reflects that the stick limit digging force is limited by this single factor; specifically, the majority of the initial bucket cutting phase is limited by small chamber locking of the boom, but as the digging operation proceeds, the digging performance becomes limited only by the full potential of the stick cylinder. In the above matching analysis, a situation arises in which the measured digging resistance near the maximum digging depth is greater than the stick digging force; this indicates that there may come a point at which digging with the stick alone is not possible, making it necessary to add the bucket to complete the digging operation.

Because the digging resistance is greater than the theoretical digging force, which mostly occurs near the maximum digging depth, the combined analysis of Figs. 9 and 10 indicates that the bucket digging force near the maximum digging depth is limited mainly by large chamber locking of the stick cylinder and small chamber locking of the boom, and the bucket full play is larger than the stick big cavity locking, indicating that the bucket mechanism is weaker than the bucket bar mechanism; Whereas the stick digging force is limited mainly by small chamber locking of the boom and full utilisation of the bucket cylinder, and the stick fully developed than the boom small cavity locking, indicating that the bucket rod mechanism is weaker than the boom mechanism. The common feature is that near the maximum digging depth, the bucket and stick digging forces are both limited by small chamber locking of the boom.

### Compound digging force limiting factor matching

**Figure 11:**
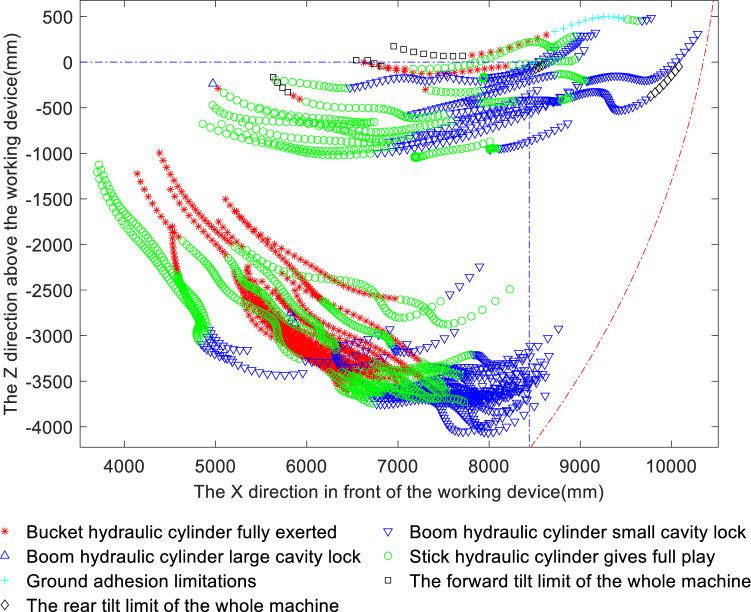
Distribution of limiting factors for the compound digging force.

Figure 11 shows the distribution of the limiting factors of the compound digging force. A comparative analysis of Figs. 9 and 10 shows that the limiting factors of bucket digging and compound digging are similar, while there is essentially only a single limiting factor of stick digging. Analysis of Fig. 11 and Table 3 shows that the proportions of instances in which the major limiting factors for compound digging are the cylinder capacities themselves are 26.2% for the bucket cylinder and 37.4% for the stick cylinder, which means that the total percentage of the time that the engine power is fully utilised is 63.6%. In addition, the full play of the stick is greater than that of the bucket, indicating that the stick mechanism is weaker than the bucket mechanism during compound digging. Among the instances in which the utilisation of the engine power is limited, the proportion of boom small chamber locking is as high as 34.4%, while the proportion of boom large chamber locking, ground adhesion and forward and rearward tilting of the whole machine is 2.0% in total, indicating that the latter factors have little effect on the compound digging force. However, the occurrence percentage of small chamber locking of the boom is too large, causing the bucket hydraulic cylinder and stick hydraulic cylinder to be underutilised, which indicates that the components of the working device are not well matched.

A comparative analysis of Figs. 9, 10 and 11 shows that all three are excessively limited by the small chamber of the boom cylinder. Therefore, to make full use of the digging capacity of the bucket and stick mechanisms, improve the matching characteristics of the various mechanisms of the working device and the overall unit, and increase the utilisation of the engine power, consideration can be given to increasing the cross-sectional area of the boom chamber, lengthening the force arm of the boom hydraulic cylinder, or increasing the locking pressure of the boom hydraulic cylinder to improve the locking capacity of the boom chamber. In this way, the bucket digging force, stick digging force and compound digging force can all be increased, allowing them to overcome greater digging resistance and thereby improving the digging performance of the excavator.

## Conclusions

The force matching indicates: the theoretical digging force is more than 80% than the measured digging resistance, and its size distribution is consistent with the measured digging resistance distribution, and it is in the cutting loading stage, most of the theoretical digging force is close to the measured digging force, and most of the excess is between 1 and 1.1 times, indicating that the theoretical digging force can better characterize the digging capacity. The main reason that the limit digging force of bucket is larger than the compound digging force is that the existing compound digging force model fails to consider the influence of resistance moment.

The characteristics of the maximum digging resistance working device show that: in the cutting and loading stage of the trenching operation, when the digging resistance in highest, the oil pressure in the small chamber of the boom cylinder is the highest and is mainly responsible for the locking of the boom cylinder, whereas the oil pressures in the large chambers of the stick cylinder and bucket cylinder are also high and are mainly responsible for generating the active force of the hydraulic cylinders. In the full-load lifting stage, the oil pressure in the large chamber of the boom cylinder is high, while the oil pressure in the small chamber is almost not involved in operation; meanwhile, the bucket cylinder and stick cylinder have similar oil pressures in their large and small chambers, although the oil pressure in the large chamber of the bucket cylinder is greater than that in the small chamber, whereas the oil pressure in the small chamber of the stick cylinder is greater than that in the large chamber. Accordingly, the bucket cylinder exerts a greater digging force than the stick cylinder in normal compound digging operations.

The matching of the limiting factors showed that: bucket digging is similar to compound digging, while stick digging has a single limiting factor. In bucket digging, the bucket mechanism is weaker than the stick mechanism, while the stick mechanism is weaker than the boom mechanism in stick digging, and the stick mechanism is weaker than the bucket mechanism in compound digging. All three groups show that the locking limit of boom hydraulic cylinder is too high, which indicates that the insufficient locking ability of boom makes the excavating ability not fully developed. To adapt to the deeper digging conditions of the trenching operation, consideration can be given to increasing the cross-sectional area of the boom chamber and the length of the force arm or increasing the locking pressure of the boom hydraulic cylinder to improve the locking capacity of the boom chamber, thus improving the digging performance of the excavator as well as the utilisation of the engine power.

## Data Availability

The datasets analysed during the current study are not publicly available because the excavator models tested were experimental models for the company. However, they are available from the corresponding author on reasonable request.
